# HIV-1 Tat-Induced Microgliosis and Synaptic Damage via Interactions between Peripheral and Central Myeloid Cells

**DOI:** 10.1371/journal.pone.0023915

**Published:** 2011-09-02

**Authors:** Shao-Ming Lu, Marie-Ève Tremblay, Irah L. King, Jin Qi, Holly M. Reynolds, Daniel F. Marker, John J. P. Varrone, Ania K. Majewska, Stephen Dewhurst, Harris A. Gelbard

**Affiliations:** 1 Center for Neural Development and Disease, University of Rochester School of Medicine and Dentistry, Rochester, New York, United States of America; 2 Child Neurology Division, Department of Neurology, University of Rochester School of Medicine and Dentistry, Rochester, New York, United States of America; 3 Department of Neurobiology and Anatomy, University of Rochester School of Medicine and Dentistry, Rochester, New York, United States of America; 4 Center for Visual Science, University of Rochester School of Medicine and Dentistry, Rochester, New York, United States of America; 5 Department of Laboratory Medicine and Pathology, University of Rochester School of Medicine and Dentistry, Rochester, New York, United States of America; 6 Department of Microbiology and Immunology, University of Rochester School of Medicine and Dentistry, Rochester, New York, United States of America; 7 Department of Psychiatry, University of Wisconsin, Madison, Wisconsin, United States of America; 8 Trudeau Institute, Saranac Lake, New York, United States of America; 9 North Shore University Health System Research Institute, Evanston Hospital, Evanston, Illinois, United States of America; 10 Diffinity Genomics, West Henrietta, New York, United States of America; Boston University School of Medicine, United States of America

## Abstract

Despite the ability of combination antiretroviral treatment (cART) to reduce viral burden to nearly undetectable levels in cerebrospinal fluid and serum, HIV-1 associated neurocognitive disorders (HAND) continue to persist in as many as half the patients living with this disease. There is growing consensus that the actual substrate for HAND is destruction of normal synaptic architecture but the sequence of cellular events that leads to this outcome has never been resolved. To address whether central vs. peripheral myeloid lineage cells contribute to synaptic damage during acute neuroinflammation we injected a single dose of the HIV-1 transactivator of transcription protein (Tat) or control vehicle into hippocampus of wild-type or chimeric C57Bl/6 mice genetically marked to distinguish infiltrating and resident immune cells. Between 8–24 hr after injection of Tat, invading CD11b^+^ and/or myeloperoxidase-positive leukocytes with granulocyte characteristics were found to engulf both microglia and synaptic structures, and microglia reciprocally engulfed invading leukocytes. By 24 hr, microglial processes were also seen ensheathing dendrites, followed by inclusion of synaptic elements in microglia 7 d after Tat injection, with a durable microgliosis lasting at least 28 d. Thus, central nervous system (CNS) exposure to Tat induces early activation of peripheral myeloid lineage cells with phagocytosis of synaptic elements and reciprocal microglial engulfment of peripheral leukocytes, and enduring microgliosis. Our data suggest that a single exposure to a foreign antigen such as HIV-1 Tat can lead to long-lasting disruption of normal neuroimmune homeostasis with deleterious consequences for synaptic architecture, and further suggest a possible mechanism for enduring neuroinflammation in the absence of productive viral replication in the CNS.

## Introduction

Despite the ability of cART to efficaciously suppress replication of HIV-1, at least one-half of virus-infected persons develop HIV-1 associated neurocognitive disorders (HAND) [1], [2]. This raises the question of how neurologic disease remains manifest, albeit in a milder, more indolent form despite effective control of HIV-1 replication with cART. Furthermore, how can HIV-1 affect normal synaptic communication between neurons in different brain regions [3], considering vanishingly low copy numbers of the virus in patients treated with cART? Because neuropathologic findings associated with HIV-1 infection of the CNS are usually obtained from postmortem tissue and cannot provide information about antecedent interactions between HIV-1, populations of immune effector cells and neurons, we performed *in vivo* modeling experiments to help answer these questions.

Microglial activation, multinucleated giant cells, and gliosis due to astrocytic hypertrophy are considered nonspecific hallmarks of HIV-1 infection and inflammation in the CNS [4]. In contrast, the severity of premortem neurologic disease has been correlated with loss of synaptic complexity, as well as macrophage burden in the CNS [5]–[8]. An early investigation of these neuropathologic events by Jones et al. [9] demonstrated infiltration of neutrophils one day after intracerebroventricular administration of either single or repeated doses of Tat into rats, followed by macrophages and lymphocytes 6 d later. They noted astrocytosis, apoptotic cells, ventriculomegaly and a decrease in hippocampal glutamate∶GABA ratios, leading to the conclusion that inflammatory cell infiltration, glial activation and neurotoxicity could be triggered by the presence of Tat in the CNS. Similarly, we have shown that acute exposure to Tat can induce dramatic and durable morphologic changes in rough endoplasmic reticulum (RER) and mitochondria, both *in vitro* and *in vivo* [9]. These changes are similar to what is observed in postmortem brain tissue of patients with HAND [10] - prompting us to investigate the sequence of neuroimmune events that occurred after stereotactic injection of Tat *in vivo*. One of the key questions we hoped to address through these experiments was the relative contribution of infiltrating myeloid cells versus brain-resident microglia to destruction of normal synaptic architecture [11]–[14].

For this study, we have elected to use HIV-1 Tat in a simplified experimental model of HAND for the following reasons: (1) patients with undetectable viral loads (and hence a vanishingly small amount of gp120 present in budding virions from productively infected cells in the CNS) still have HAND [15]; (2) ample data exists to validate the presence of Tat in both lymph and CNS compartments with an equal ability to induce transactivation [16]; (3) more “pathophysiologically” faithful murine models of HAND such as the CD34-NSG HIV-1 infected mice described in our recent publication by Dash et al. [17] still require technically difficult experimental maneuvers to create animals in sufficient quantity for *in vivo* experiments described in this paper; and (4) perhaps most importantly, we needed to temporally and spatially control the nature of a single exposure to Tat in the CNS in order to investigate the effects of persistent immune activation on normal synaptic architecture.

Using mice transgenic for fluorescently labeled peripheral and central mononuclear phagocytes, we now report that exogenous application of a single dose of Tat into the CNS induced acute infiltration of peripheral mononuclear phagocytes and myeloperoxidase-positive granulocytes, starting as early as 8 hours after the injection. We further show, for the first time, infiltrating inflammatory leukocytes engulfed dendritic spines and microglial processes. In contrast, microglia near the site of Tat exposure remained in a hypertrophied, arborized phenotype for at least 28 days, but were nevertheless found to acutely engulf mononuclear phagocytes as well as synaptic structures including axon terminals, dendrites, and dendritic spines. Taken together our data suggest a single exposure to the HIV-1 regulatory Tat protein can transiently increase infiltration of inflammatory peripheral leukocytes, resulting in microgliosis accompanied by disruption of normal synaptic architecture.

## Results

Preclinical *in vivo* models for HAND include: the administration of HIV-1 neurotoxic gene products, gp120 and Tat into the CNS; introduction of HIV-1 infected mononuclear cells into the CNS; transgenic mouse and rat models (e.g., GFAP-gp120 and GFAP-Tat), and *in vivo* simian immunodeficiency virus (SIV) infection of the CNS, SIVE [9], [18]–[24]. These have collectively recapitulated at least some neuropathologic findings found in patients with HAND [4] - including microglial activation, infiltration of perivascular macrophages and astrocytosis. However, the persistence of HAND in the face of low or absent viral loads has forced us to reinvestigate why the presence of secretory neurotoxins in the neuropil or synapse alone cannot fully explain the neuropathogenesis of HAND.

### Time course of neuroinflammation following stereotactic injection of Tat

**Figure 1:**
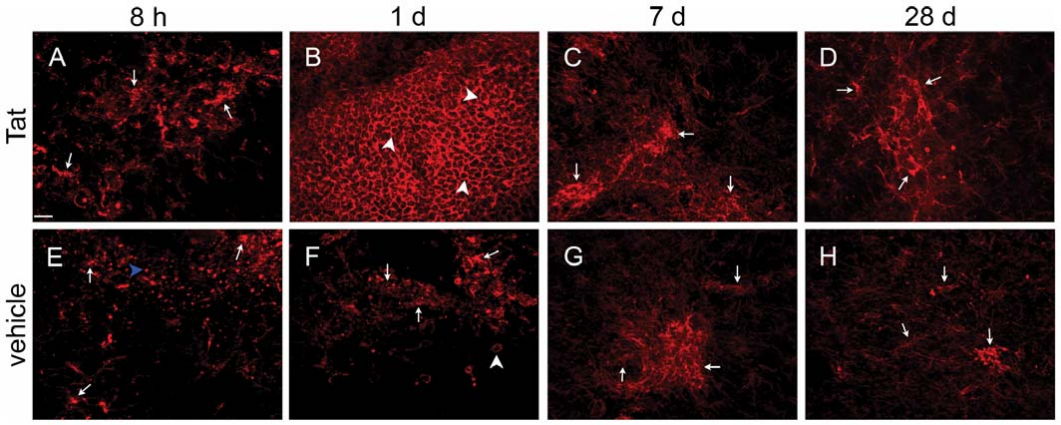
HIV-1 Tat causes enduring microglial activation and transient leukocyte infiltration into hippocampal neuropil. Both microglia and leukocytes were visualized with CD11b^+^ and they could be distinguished by their morphology because infiltrating leukocytes had round or ovoid morphology whereas microglia had ramified, often hypertrophied processes. Wild-type mice received either Tat (top panels, n = 3) or saline control vehicle (bottom panels, n = 3) injection into the right hippocampus and were sacrificed 8 hours (Panels A and E), 1 (Panels B and F), 7 (Panels C and G) and 28 (Panels D and H) days later, and hippocampal tissue sections processed for CD11b immunocytochemistry as described in Methods. All microscopic fields depicted here are directly adjacent to the injection site. White arrows point to examples of ramified microglia and white arrowheads point to examples of round or ovoid infiltrating leukocytes. The blue arrowhead in E points to a microglial cell fragment that was frequently seen in both the Tat and saline treatment groups 8 hours after injection. These cellular fragments typically appeared as isolated globules scattered in and around the injection site but were much smaller than leukocytes. Scale bar = 16 µm for all micrographs.

We elected to investigate the effects of acute hippocampal exposure to HIV-1 Tat because a large body of literature using both rats and mice has demonstrated robust Tat-associated changes in synaptic architecture in this brain region as well as behavioral consequences [25]–[29]. Initial experiments with higher *in vivo* doses (25 µg/5 µl) of Tat used in a previously published report [30], resulted in significant neuronal loss and cavitation at the injection site. This prompted us to reduce the dose of Tat by 40% (i.e. 15 µg/5 µl) in order to investigate potential effects of Tat on synaptic architecture as well as changes in populations of immune effector cells. Thus, we injected Tat or vehicle control into the hippocampus of wild-type mice and performed a time course experiment, sacrificing mice 8 hrs, 1 d, 7 d and 28 d after injection. To survey leukocyte responses to Tat, we performed immunocytochemical studies to assess the relative expression of leukocyte integrin CD11b around the injection site. Even though CD11b does not differentiate between leukocyte subtypes [31], we were able to distinguish between infiltrating leukocytes with a round morphology and brain-resident microglia with a ramified morphology,

Examination of hippocampal tissue around the site of Tat injection revealed that both Tat and saline caused microglial activation and fragmentation adjacent to the needle track at 8 hours following injection, with few infiltrating leukocytes (Figure 1, Panels A and E). However, 1 d after the hippocampal inoculation, we detected a striking number of infiltrating leukocytes entirely encasing the center of the injection site, reminiscent of a granulomatous response, in mice that received Tat (Fig. 1B), but not in animals that received vehicle (Fig. 1F). Indeed, 1 d after vehicle treatment only a few leukocytes infiltrated the injection site with some microglia that remained in a surveillance phenotype (i.e. ramified morphology). At a later time point (7 days after injection), both treatments induced microgliosis. However, Tat treatment induced a much larger territory of microgliosis that extended into adjacent fields (data not shown), in contrast to control vehicle treatment. Finally, at an extended time point (28 days following injection), Tat treatment resulted in persistent microgliosis whereas control vehicle treatment demonstrated scattered cells with increased CD11b expression, possibly labeling dendritic cells, but no evidence of reactive microglial morphology. To further investigate this finding, we performed additional studies with both antibodies to CD11b and ionized calcium binding adapter molecule-1 (Iba-1) [32]. 28 d after exposure to Tat we observed reactive microglial morphology both by CD11b expression (Figure S1, Panel D) and Iba-1 expression (Panel B), while exposure to vehicle control 28 d later revealed fairly normal ramified morphology of microglia by CD11b, with a single very bright CD11b cell that may reflect a dendritic cell (Panel C) and Iba-1 (Panel A), respectively.

### Creation of chimeric models to investigate the role of peripheral vs. central immune responses to stereotactic injection of Tat

Because maximal infiltration of peripheral leukocytes occurred 24 hrs after Tat injection, we chose this time point to investigate whether there is a differential contribution of central and peripheral immune effector cells to the neurotoxic effects of Tat in the hippocampus. To do this, we generated bone marrow chimeras between CD45.1 mice and mice that express green fluorescent protein (GFP) under the control of the CX3CR1 promoter. In these mice, circulating monocytes, subsets of NK and dendritic cells, and microglia all express GFP [33], [34]. We then examined the ability of Tat to induce leukocyte infiltration in comparison to control vehicle. We used three experimental groups: (1) bone marrow from CX3CR1^+/−^ heterozygotes engrafted into a congenic CD45.1 host, (2) bone marrow from CX3CR1^+/+^ homozygotes engrafted into a CD45.1 host and (3) CD45.1 marrow engrafted into a heterozygous CX3CR1^+/−^ host.

We reasoned that CD45.1 hosts engrafted with CX3CR1-GFP^+/−^ marrow would allow us to assess the contribution of GFP^+^ peripheral myeloid subsets of leukocytes to neuroinflammation, which would appear as non-process-bearing amoeboid GFP+ cells in the vicinity of the injection site for Tat. Conversely, heterozygous CX3CR1-GFP^+/−^ hosts engrafted with marrow from CD45.1 animals were used to examine the contribution of GFP^+^ microglia, which might appear as rod-like, reactive microglia or microglia with processes convergent on the injection site for Tat. Finally, CD45.1 hosts engrafted with marrow from either CX3CR1-GFP^+/−^ heterozygous or CX3CR1-GFP^+/+^ homozygous mice were used to further determine whether CX3CR1 played an integral role in recruiting peripheral leukocytes to augment Tat-induced neuroinflammation. Pilot experiments revealed that engraftment successfully repopulated precursor pools for mononuclear phagocytes after 4 weeks. Thus 4 weeks after engraftment, mice received Tat or control vehicle into the right hippocampus as described in the Materials and Methods section.

**Figure 2:**
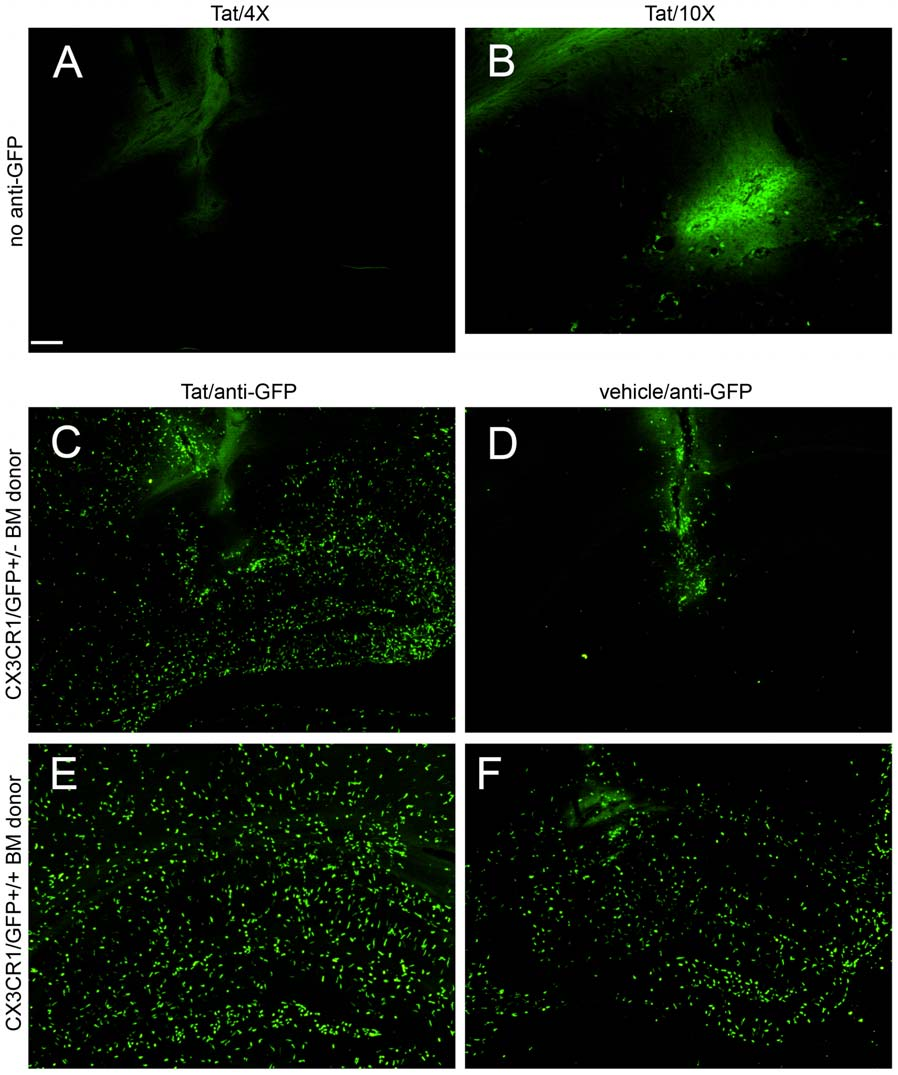
Stereotactic injection of Tat induces a differential effect on infiltrating inflammatory leukocytes depending on gene dose of CX3CR1. Montage of microscopic fields 24 hours after Tat (Panels A–C, E) or control vehicle (Panels D, F) injection into the right hippocampus of irradiation chimeras. Images in Panels A, B, C, and D were taken from CD45.1 host mice engrafted with heterozygous CX_3_CR1/GFP^+/−^ bone marrow, whereas images E and F were from CD45.1 host mice engrafted with homozygous CX_3_CR1/GFP^+/+^ bone marrow. A, C, D, E, and F were taken with a 4× objective with the same 0.9 sec exposure time and B was taken with a 10× objective with a longer exposure time (4 sec) to intensify the weak fluorescence of the GFP signal. Panel A and B depict very low expression of fluorescence from infiltrating GFP expressing leukocytes, while Panels C–F depict amplified GFP expression with anti-GFP antibody and Alexa 488 conjugated second antibody treatment. All experimental and control groups, n = 3 independent replicates. Scale bar = 100 µm for Panels A, C, D, E, and F; 40 µm for B.

We speculated that if microglia contribute to the inflammatory changes and neuronal destruction that occur after injection of Tat into the CNS, they would not remain in a resting ramified morphologic phenotype in the CX3CR1 host, but would likely assume a reactive phenotype, with a rod-like appearance, devoid of branching processes. Conversely, if peripheral leukocytes infiltrate the CNS to contribute to the initial inflammatory changes after injection of Tat, we expected to observe a large influx of green cells in the CD45.1 congenic host. However, leukocytes with a low expression level of GFP that infiltrate the parenchyma are likely to represent CX3CR1^low^ “inflammatory” monocytes, which are selectively recruited to areas of inflammation [35]. Figure 2, Panel A demonstrates that 24 hours after injection of Tat, GFP-positive cells were nearly undetectable in the hippocampus of CD45.1 host mice engrafted with heterozygous CX3CR1-GFP^+/−^ marrow. Panel B depicts the injection site, magnified 2.5×, to demonstrate that GFP^+^ cells are present but difficult to visualize without either longer exposure times or augmentation of their signal with anti-GFP antibody (used in panels C–F). These results demonstrate that infiltrating macrophages after Tat injection have low levels of CX3CR1 expression suggesting that they are derived from resting, circulating monocytes [36]. This also indicated the need to use an anti-GFP antibody to amplify the GFP signal in order to fully appreciate CNS infiltration by peripheral mononuclear cells. Thus, we used anti-GFP amplification for all further studies of irradiation chimeras that were transgenic for CX3CR1-GFP. Use of the anti-GFP antibody in an adjacent tissue section revealed a dramatic infiltrate of cells with low levels of GFP expression 24 hrs after Tat injection (Panel C), while the vehicle control resulted in infiltration of GFP^+^ cells in the region of the needle track but not in the parenchyma, suggesting that mechanical breach of the blood brain barrier alone did not account for recruitment of GFP-expressing cells in the parenchyma (Panel D).

Because of concerns that total body irradiation during the generation of CD45.1 mice engrafted with CX3CR1-GFP^+/−^ marrow would lead to damage to the blood-brain barrier (BBB), we repeated our experiments with head-shielding to prevent radiation injury to the brain [37]–[39]. Quantification of our results using one-way ANOVA with Tukey's HSD post-hoc test demonstrated that head shielding made no difference to monocyte infiltration into the hippocampus after Tat or control vehicle injection. There were 1170±177 GFP+ cells with shielding (N = 3) vs. 1271±108 GFP+ cells without shielding (N = 3)(p = 0.815) infiltrating into the hippocampus 24 hr after Tat injection and 18±12 GFP+ cells with shielding (N = 3) vs. 22+4 GFP+ cells without shielding (N = 3)(p = 1.00) after control vehicle injection (Figure S2, Panel A). However, there was a significant difference in the monocyte infiltration between Tat and control vehicle treatments (p<0.001, regardless of head shielding).

### Role of CX3CR1-GFP gene dose on leukocyte infiltration in response to Tat

To further investigate the role of CX3CR1 in the ingress of peripheral mononuclear cells, we injected Tat into the hippocampus of CD45.1 mice engrafted with homozygous CX3CR1-GFP^+/+^ marrow. Mice engrafted with homozygous CX3CR1-GFP^+/+^ marrow that received Tat (Figure 2, Panel E) had significantly greater numbers of GFP+ cells in the parenchyma (2089±96, N = 2) than CD45.1 mice engrafted with heterozygous CX3CR1/GFP^+/−^ marrow that also received Tat (1271±108, N = 3) (p<0.001, one-way ANOVA with Tukey's HSD post-hoc test) (Panel C)(Figure S2, Panel B). Interestingly, the numbers of adoptively transferred immune cells from homozygous CX3CR1/GFP^+/+^ mice that infiltrated into the CNS were also much higher in the vehicle control (1457±88, N = 2 vs. 22±4, N = 3)(p<0.001, one-way ANOVA with Tukey's HSD post-hoc test)(Panel F vs. Panel D)(Figure S1, Panel B).

**Figure 3:**
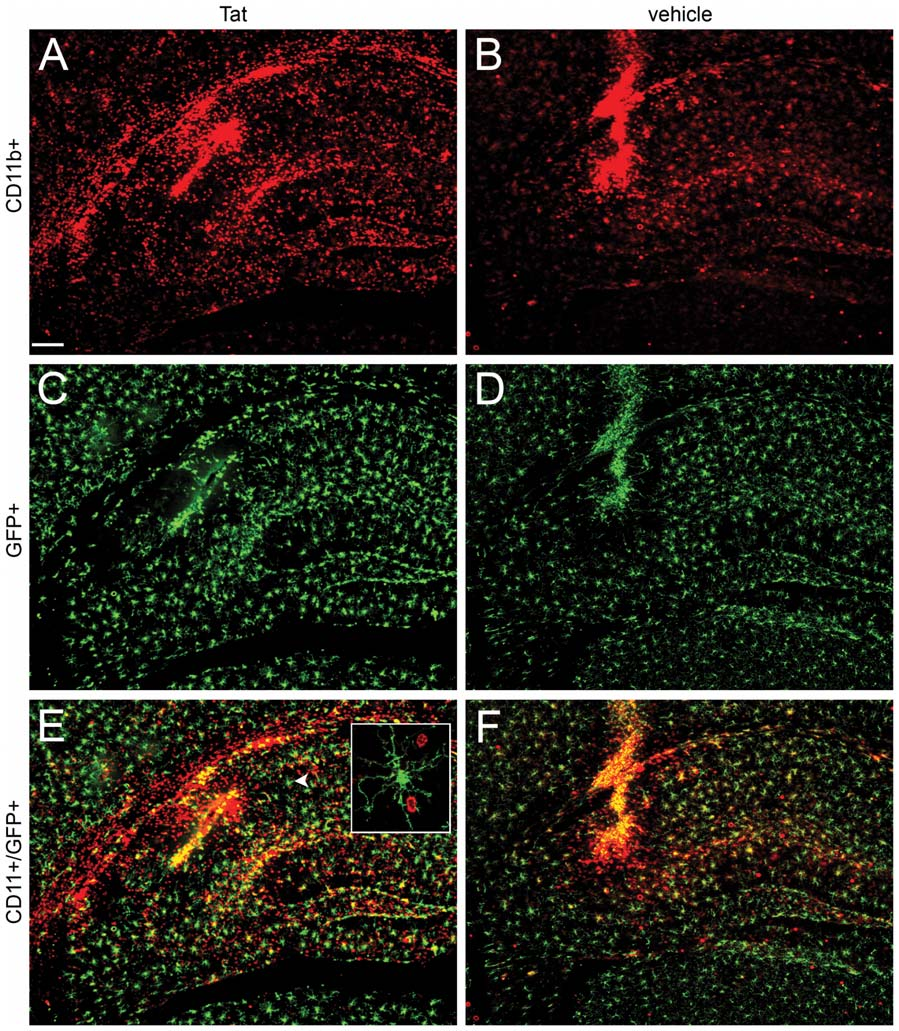
Stereotactic injection of Tat into hippocampus does not induce amoeboid microglial morphology in irradiation chimera. Montage of micrographs 24 hours after Tat (Panels A, C, and E) or control vehicle (Panels B, D, and F) injection into right hippocampus in CX_3_CR1/GFP^+/−^ hosts engrafted with congenic CD45.1 bone marrow. A and B show CD11b^+^ cells (red), C and D show GFP^+^ microglia (green) present in the same section, and E and F presents an overlay of the two images - highlighting the CD11b^+^/GFP^+^ co-expressing cells (orange) as well as the single positive cells. The inset in panel E shows a ramified GFP+ microglial cell (white arrowhead) in higher magnification and the 2 infiltrating CD11b+ peripheral leukocytes (red). All experimental and control groups, n = 3 independent replicates. Scale bar shown in Panel A = 100 µm for all figures.

**Figure 4:**
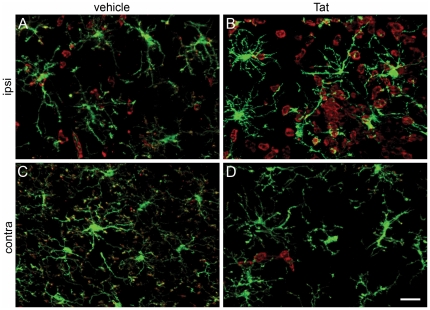
High resolution micrographs confirm lack of amoeboid microglial morphology following exposure to Tat in irradiation chimera. Montage of high resolution micrographs illustrating the microglia remain in a ramified morphology 24 hours after Tat (Panels B, D) or control vehicle (Panels A, C) injection into right hippocampus in CX_3_CR1/GFP^+/−^ hosts engrafted with congenic CD45.1 bone marrow. Shown are CD11b^+^ cells (red) and GFP^+^ microglia (green). While thickened processes are frequently observed, there is no evidence of amoeboid microglial morphology. Panels A and B were taken from an area of the hippocampus near the injection site of vehicle and Tat, respectively, and C and D were from the corresponding area of the hippocampus contralateral to the injection site of vehicle and Tat, respectively. All experimental and control groups, n = 3 independent replicates. Scale bar = 20 µm.

Next we investigated the morphology of brain-resident microglia 24 hrs after injection of Tat or control vehicle into the hippocampus of CX3CR1-GFP^+/−^ that had been engrafted with bone marrow from CD45.1 mice. As in Figure 2, we noted dramatic infiltration of CD11b^+^ leukocytes around the needle track and parenchyma in response to Tat (Figure 3, Panel A), while Panel B shows CD11b^+^ leukocyte infiltration primarily around the needle track with scattered infiltration in the parenchyma in response to control vehicle. Figure 3, Panels C and D show that microglia adjacent to the injection site and further away from the needle track in hippocampus remained in ramified morphology (Figure 3, Panel F); this is even more clearly apparent in higher magnification images (Figure 4). There was marked infiltration of CD11b^+^/GFP^−^ cells adjacent to the needle track and in hippocampus of Tat-treated (Figure 3, Panel E) relative to mice treated with vehicle alone (Figure 3, Panel F), further suggesting that infiltrating leukocytes are primarily responsible for acute inflammation following Tat injection. Analysis of tissue sections up to 500 µm from the injection site confirmed that Tat induced a differential increase in leukocyte infiltration compared to saline control vehicle (data not shown).

**Figure 5:**
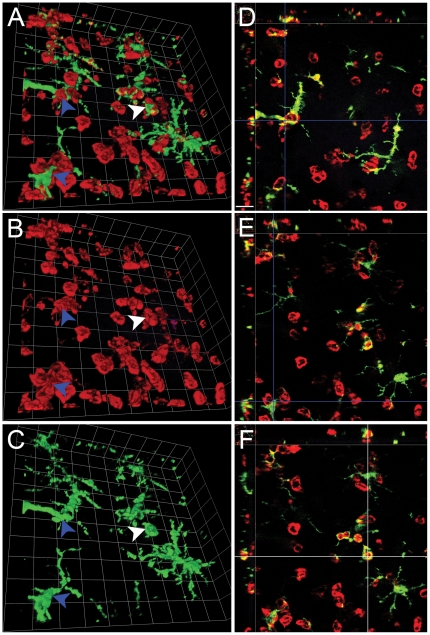
Stereotactic injection of Tat results in reciprocal engulfment of microglia and infiltrating leukocytes. Montage of higher magnification micrographs with 3D reconstruction of GFP^+^ microglia (green) interacting with infiltrating CD11b^+^ leukocytes (red) in the right hippocampus of mice injected with Tat 24 hours earlier (Panels A, B, and C) and XYZ views at different depths of the same image stack (Panels D, E, and F). Blue arrowheads points to the corresponding locations where infiltrating leukocytes engulf microglia processes. Panel A shows a combined view of CD11b (red) and GFP (green) expression, while panel B shows CD11b expression alone and panel C shows GFP expression alone. The white arrowhead points to the location where GFP^+^ microglial processes phagocytose a CD11b^+^ infiltrating leukocyte. To further illustrate the phagocytic nature of these cell-to-cell interactions, Panels D and E show the corresponding XYZ view of the location (the crossing point of the two blue cursor lines) of the top and bottom blue arrowhead in panel A, respectively. Panel F shows the XYZ view of the location (the crossing point of the two white cursor lines) of the white arrowhead in Panel A. Each side of individual grids = 14 µm for Panels A–C, and the scale bar shown in Panel D = 16 µm for D–F. n = 3 independent replicates for Tat treatment.

To further investigate the response of brain-resident microglia to Tat-induced neuroinflammation, we examined three-dimensional (3D) reconstructions of brain tissue 24 hrs after Tat injection into the hippocampus of CX3CR1-GFP^+/−^ hosts engrafted with CD45.1 bone marrow. We differentiated between microglia based on their ramified morphology, relatively uniform GFP expression and lower levels of CD11b expression when compared to infiltrating leukocytes, which were amoeboid, lacking GFP expression, frequently convergent and had higher levels of CD11b expression. Figure 5 demonstrates two examples of infiltrating leukocytes that appear to phagocytose microglia (blue arrows, Panel A) and one example of a microglial cell that appears to phagocytose leukocytes (white arrow, Panel A). Panels B and C show CD11b and GFP expression in these cells, respectively. Panels D and E show the corresponding cross-sectional (XYZ) view (see Methods: Image Analyses) to help validate leukocyte phagocytosis of microglia and conversely Panel F shows the same 3D view of microglial phagocytosis of leukocytes. When phagocytosis occurred in hippocampal parenchyma, CD11b^+^ leukocyte fragments were clearly visible inside GFP^+^ microglia or vice-versa.

### Role of granulocyte infiltration in response to stereotactic injection of Tat

**Figure 6:**
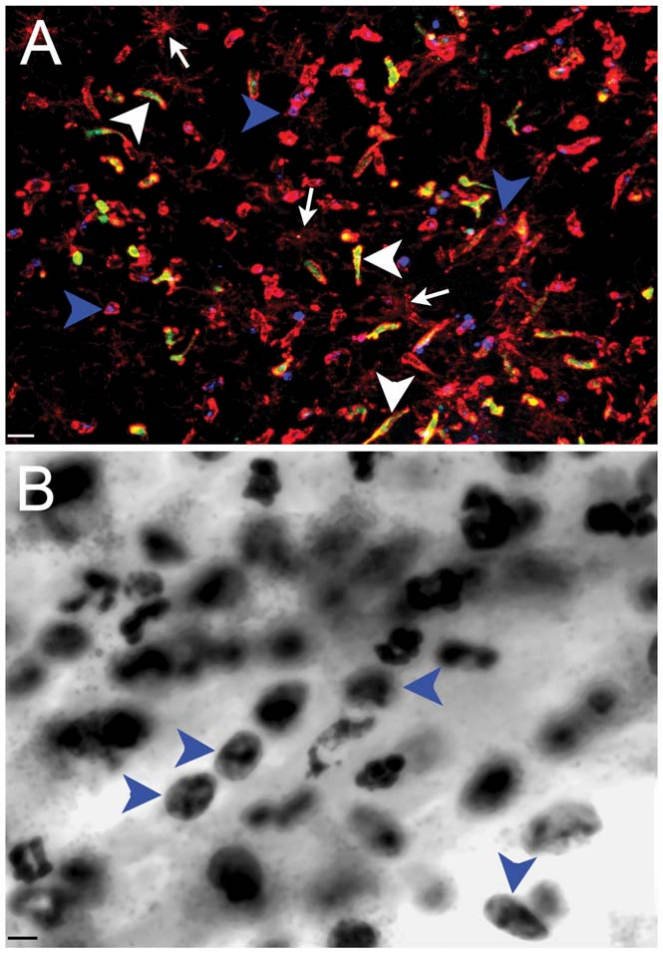
Stereotactic injection of Tat into hippocampus induces Infiltration of polymorphonuclear cells 24 hours later. Panel A illustrates combined immunofluorescence staining of CD11b (red), MPO (Blue), Iba-1 (white), and GFP (green) cells in a representative tissue section from a congenic CD45.1 host engrafted with homozygous CX3CR1/GFP^+/+^ marrow that received Tat. Blue arrowheads point to CD11b^+^/MPO^+^/GFP^−^ infiltrating leukocytes, white arrowheads point to CD11b^+^/MPO^−^/GFP^+^ infiltrating leukocytes, and white arrows point to CD11b^+low^/MPO^−^/GFP^−^ ramified microglia. Panel B illustrates a 100× oil-immersion image from H&E staining of an adjacent section from Panel A and blue arrowheads point to polymorphonuclear cells. Scale bar = 16 and 8 µm for Panels A and B, respectively. n = 3 independent replicates for Tat treatment.

Jones [9] reported that neutrophil infiltration occurred 24 hrs after intraventricular administration of Tat, and we also noted the presence of round cells adjacent to the injection site that were CD11b^+^. We performed additional immunocytochemical studies with a myeloperoxidase marker (MPO) to identify granulocytes [40], using CNS tissue from congenic CD45.1 mice engrafted with homozygous CX3CR1-GFP^+/+^ donor marrow because we reasoned that this genotype would increase the number of infiltrating inflammatory leukocytes, collected 24 hrs after injection with Tat. Figure 6 shows a single representative field of right hippocampus adjacent to the injection site with four-color labeling of antigens and GFP that identify mono- and polymorphonuclear populations of infiltrating leukocytes as well as microglia. Panel A demonstrates that a substantial proportion of infiltrating leukocytes were CD11b^+^/CX3CR1-GFP^−^ cells that expressed MPO (blue; blue arrowheads). There was a similar degree of co-localization between GFP+ and CD11b+ infiltrating leukocytes (white arrowheads). In contrast, white arrows in Panel A identify CD11b^+low^/MPO^−^/GFP^−^ ramified microglia.

To validate our interpretation that a substantial population of round cells infiltrating the CNS 24 hours after the hippocampal injection of Tat were indeed granulocytes, a blinded neuropathologist (M.J.) examined H&E stained slides of hippocampus and confirmed the presence of polymorphonuclear leukocytes in mice that received Tat. This was consistent with our data that demonstrated cells with neutrophil morphology only in tissue sections from mice that had received Tat (Fig. 6B, blue arrowheads), but not in vehicle-treated controls (data not shown). In chimeric mice that received head shielding during irradiation, the majority (64%) of the total number of leukocytes (GFP+ and MPO+ cells) that infiltrated the hippocampus in response to Tat appeared to be granulocytes (2110±379 MPO+ cells 24 hr after Tat injection vs. 32±10 MPO+ cells 24 hr after vehicle control)(Figure S2, Panel C).

### Microglial-neuronal contact in response to stereotactic injection of Tat

**Figure 7:**
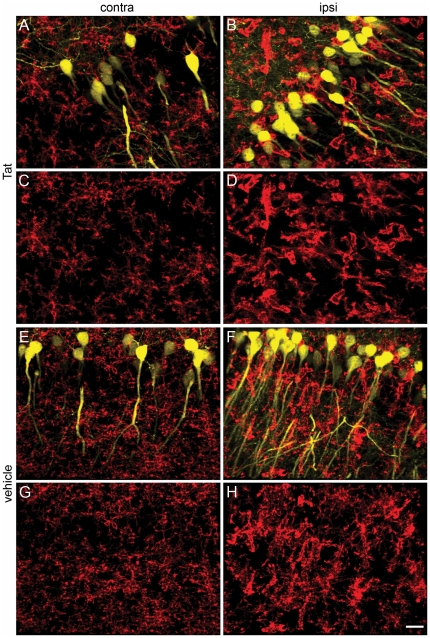
Exposure to Tat alters microglial morphology and spatial relationships with neurons. Montage of thy-1 YFP^+^ neurons (yellow) and CD11b^+^ (red) 24 hr after Tat (Panels B, D, F, H) or control vehicle (Panels A, C, E, G) injection into right hippocampus. Images in Panels A–D are from the CA1–CA2 region about 1 mm away from the vehicle (A, C) or Tat (B, D) ipsilateral to the injection site. Images in Panels E–H are taken from the CA1 region of the contralateral hippocampus of mice injected with vehicle (E, F) or Tat (G, H). Scale bar = 20 microns. Note that CD11b+ microglia (red) in control vehicle treated hippocampi exhibited a normal resting ramified morphology with comparatively thin processes that bear many small appendages (A, C, E, and G), whereas CD11b+ microglia in Tat-treated hippocampi have an activated morphology with much thicker processes and enlarged cell bodies. Some of the hypertrophied processes wrap around YFP^+^ dendrites and cell bodies. Note that the number of YFP^+^ neurons varies between mice. For quantitation (see Results and Figure S2, Panel D), we normalized our data relative to YFP expression in each mouse. For Tat and control groups, n = 3 independent replicates.

Additionally, we examined the intricate spatial relationships between CD11b^+^ ramified microglia and neuronal dendrites. To do this, we took advantage of thy1-YFP transgenic mice, in which the YFP reporter gene is expressed in hippocampal and cortical neurons. We injected Tat or saline into the right hippocampus of these animals, and 24 hrs later examined the site of injection using immunocytochemical staining methods. Even though microglia retained a ramified morphology after stereotactic injections, they developed markedly hypertrophied processes in response to Tat treatment (Figure 7 Panels B and D ipsilateral, and F and H contralateral to the injection) compared to vehicle treatment (Figure 7 A and C ipsilateral, and E and G contralateral to the injection).

**Figure 8:**
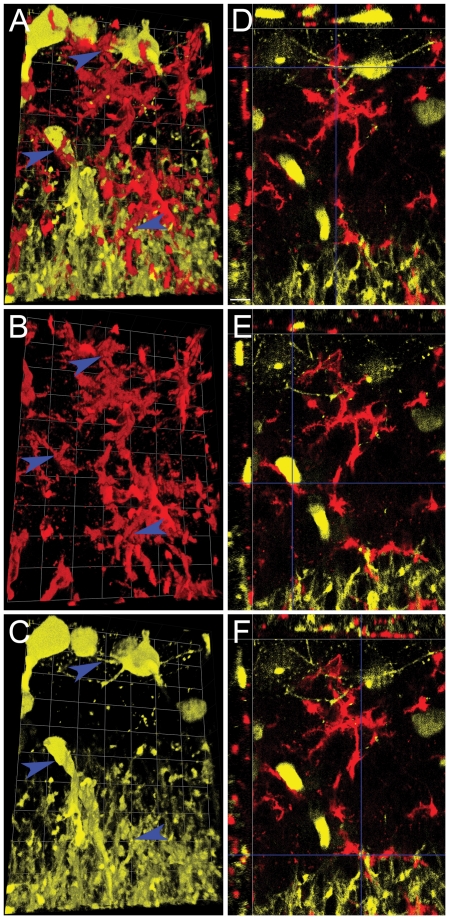
Stereotactic injection of saline control does not induce engulfment of dendrites. Montage of 3D reconstruction micrographs of a representative tissue section from right hippocampus of YFP_H_ mice that received saline control vehicle and were sacrificed 24 hours later (n = 3 replicates). Panels A–C depict a 3D reconstruction of CD11b^+^ microglia (red) interacting with YFP^+^ pyramidal neurons (yellow), while Panels D–F show XYZ views at different depths in the same image stack. Blue arrowheads point to cell-to-cell contact between microglia and neuronal processes. These points of contact are illustrated in the XYZ view in Panels D and F (crossing point of the two blue cursor lines). Panel E shows an XYZ view of a neuronal soma contacting a microglial process (crossing point of the two blue cursor lines). Each side of the individual grid = 14 µm in Panels A, B, and C, and the scale bar in Panel D = 16 µm for Panels D–F.

**Figure 9:**
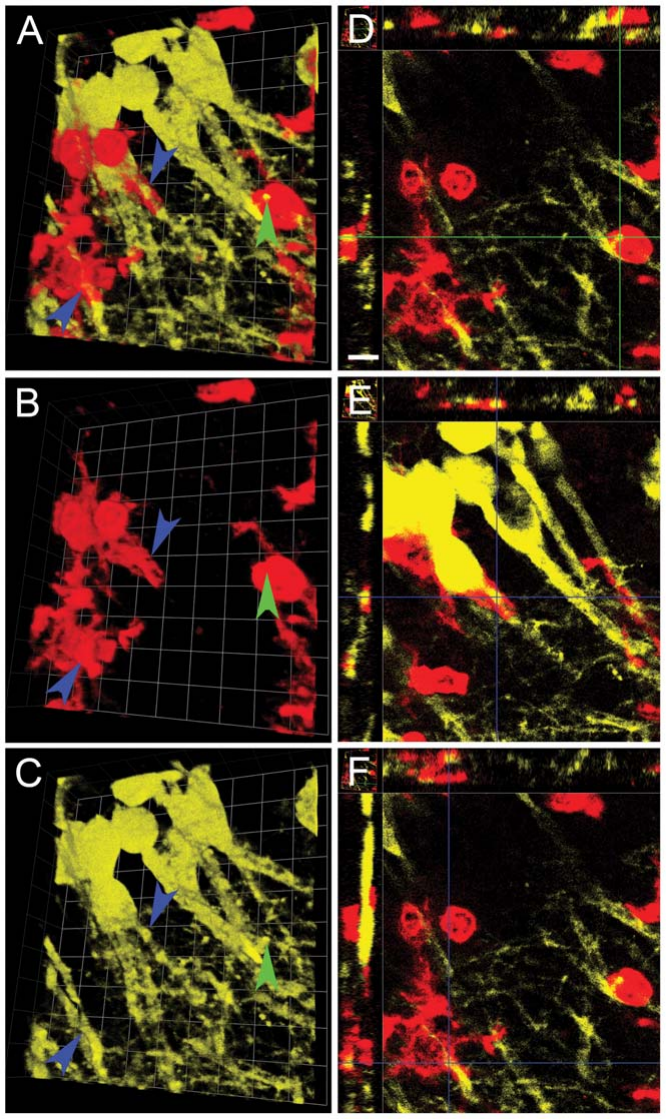
Stereotactic injection of Tat induces infiltrating leukocyte engulfment of dendrites. This is a montage of 3D reconstruction micrographs of a representative tissue section of right hippocampus of YFP_H_ mice that received Tat and were sacrificed 24 hours later (n = 3 independent replicates). As in Figure 7, Panels A–C depict a 3D reconstruction of CD11b^+^ microglia (red) and infiltrating leukocytes (also red) interacting with YFP^+^ pyramidal neurons (yellow) while Panels D–F show XYZ views at different depths in the same image stack. Green arrowhead points to an infiltrating leukocyte phagocytosing a dendritic process from an YFP^+^ pyramidal neuron, which is depicted in the XYZ view in Panel D (crossing point of the two green cursor lines). In contrast, blue arrowheads identify where microglia processes appear to ensheath neuronal processes. Panels E and F depict the XYZ view of this microglial ensheathment of a neuronal process (crossing point of the two blue cursor lines). Each side of individual grid = 9 µm in Panels A–C, and scale bar in Panel D = 10 µm for Panels D–F.

In Figure 8, 3D views of saline-exposed hippocampus (collected at 24 hrs following intracranial injection) are depicted in Panels A–C and cross-sectional confocal-like XYZ views are depicted in the corresponding Panels D–F. The extensive and intricate neuritic processes emanating from neuronal soma can be readily appreciated, as can the fact that there are almost no CD11b^+^ infiltrating leukocytes adjacent to neuritic processes, and that CD11b^+^ ramified processes of microglia surround or even ensheath YFP-expressing hippocampal neurons. Points where cell-to-cell contact between microglia and neurons occur are illustrated in 3D and their corresponding XYZ view with blue arrowheads. In striking contrast, Figure 9 demonstrates similar views of the hippocampus from Tat-exposed animals, also collected at 24 hrs following intracranial injection. The images reveal infiltrating CD11b^+^ round leukocytes, one of which is phagocytosing a YFP^+^ dendritic spine (green arrowhead, Panel A), while CD11b^+low^ microglia are ensheathing neuronal processes (blue arrowheads, Panel A), 24 hrs after injection of Tat into hippocampus. From these data, we concluded that infiltrating leukocytes with an inflammatory phenotype not only attack brain-resident microglia but phagocytose synaptic elements in response to Tat-induced neuroinflammation.

We next quantified the amount of contact between CD11b^+^ structures, leukocytes and microglia, and YFP^+^ neuronal structures. This was measured as the intersecting volume between the two from 3D image sets at least 500 µm away from the stereotactic injection site from 4 Tat treated (24 hr) and 3 vehicle treated (24 hr) mice using Volocity 3DM software. Because of the variability of hippocampal YFP expression between mice, we normalized the intersecting volume between CD11b^+^ and YFP^+^ structures by dividing it with the total YFP^+^ volume from each individual image set. Morphometric quantification of these volumes demonstrated a 41% increase in contact between CD11b^+^ leukocytes and microglia with neuronal structures after Tat compared to vehicle treatment (2.41×10^−6^±5.3×10^−7^ vs. 1.71×10^−6^±4.94×10^−7^, P<0.01 by T-test)(Figure S2, Panel D).

### Immunoelectron microscopy of neuronal-microglial and neutrophil interactions in response to stereotactic injection of Tat

**Figure 10:**
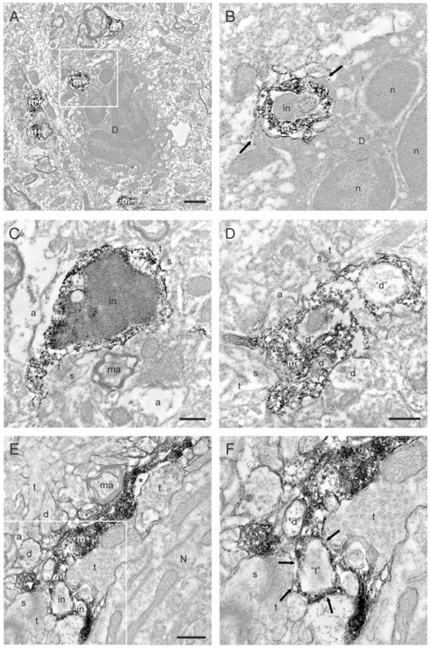
Tat alters ultrastructural relationships between polymorphonuclear granulocytes, microglia and neuronal elements including synapses in the cerebral cortex of CX_3_CR1^+/−^ mice. This is a montage of electron micrographs 24 hrs (Panels A–D) or 7 d (Panels E, F) after Tat injection (n = 3 independent replicates). Panels A and B show a polymorphonuclear granulocyte (pmn) cell body in apposition to a microglial process. Panel B presents a higher resolution view of the boxed region in Panel A Two processes arising from the degenerating polymorphonuclear granulocyte (arrows) surround an Iba-1 immunopositive microglial process (m+) with a cellular inclusion (in). Panel C presents another example of microglial process with a cellular inclusion. In both Panels B and C, the cellular inclusion appears as engulfed material from degenerating neurons. In Panel D, a microglial process contains three cellular inclusions resembling profiles of dendrites (“d”). Direct contacts with other synaptic elements including dendritic spines (s) and axon terminals (t) are also observed. Panel E shows a microglial process displaying a cellular inclusion resembling an axon terminal (“t”; arrows), as well as cellular inclusions resembling a dendrite, suggesting their phagocytic engulfment by microglia after HIV infection. Panel F presents a higher resolution view of the boxed region in Panel E. a, astrocytic process; d, dendrite; ma, myelinated axon; n, nucleus; N, neuron; *, extracellular space; scale bars, 1 µm (Panel A) and 0.5 µm (Panels C–E).

Because our light microscopic data could not fully resolve whether apposition of microglia around neuritic processes resulted in engulfment of synaptic elements in response to Tat, we performed ultrastructural studies of ionized calcium binding adaptor molecule 1 (Iba1) immunostained tissue sections collected from the cortex of CX3CR1-GFP^+/−^ mice 24 hr or 7d after stereotactic injection of Tat (Figure 10). We reasoned that high numbers of inflammatory granulocytes infiltrate brain parenchyma 24 hrs after exposure to a single dose of Tat, but would be largely absent 6 d later. These two time points would allow us to distinguish whether there were any qualitative differences in ultrastructural relationships between microglia or neutrophils and neurons. For these studies, we used cortex, where similar inflammatory changes in populations of infiltrating leukocytes and microglia were observed (data not shown), and Iba-1 staining was used to identify microglia. 24 hrs after Tat injection, we observed an obvious increase in the prevalence of degenerating neurons (versus saline controls) with the caveat that polymorphonuclear granulocytes were frequently observed at the 24 hrs time point, but not observed at 7 d. Some of the neuronal processes were found surrounded by Iba1 immunopositive microglial processes, which in turn were engulfed by granulocytes with a typical multinucleated appearance (Inset box, Panel A, higher magnification, Panel B) [41]. We also observed an increase in the prevalence of cellular inclusions inside microglia (versus saline controls), sometimes resembling synaptic elements (dendritic spines and dendrites, axon terminals)(Panels C, D). 7 d after Tat injection, we frequently observed cellular inclusions resembling synaptic elements within microglia, but were unable to see any polymorphonuclear granulocytes with or without inclusions resembling synaptic elements or microglial processes in our ultrastructural survey.

## Discussion

In this study, we stereotactically injected a single dose of HIV-1 Tat into murine hippocampus and cortex to study the temporal sequence of events that leads to the neuroinflammation and abnormal synaptic architecture associated with HAND. Introduction of Tat into the CNS resulted in acute infiltration of leukocytes, adjacent and remote to the injection site, with engagement of microglia in cell-to-cell contact between synapses and invading leukocytes with inflammatory phenotypes. A pragmatic caveat to note is that we used an *in vivo* dose of Tat (3 µg/µl) that is ≥1000× the dose commonly used in *in vitro* experiments (1–100 nM). There is no reliable way to measure the actual concentration of Tat in the extracellular space in the CNS because no successful ELISA strategy exists and because native Tat is incredibly sensitive to oxidation. Thus, there are no published reports of local Tat concentrations in the brains of patients with HAND, but ample evidence of Tat transcripts in HAND brains [16], [42]. When cell lines are transfected with Tat, supernatants have extraordinary neurotoxicity, with a theoretical LD_50_ in the femtomolar range, far too small to be detected on a gel (Avi Nath, personal communication). When Tat is produced for experimental use, it is made and stored under reducing conditions, which are thought to represent the extracellular environment in the CNS. However, Tat is also extraordinarily sticky, and between its marked sensitivity to oxidation and ability to bind even to silanized glass surfaces (as in the syringe barrel and needle used in stereotactic injections), we used Tat_1–72_ at a pharmacologic concentration (i.e. 3 µg/µl in our study) to abrogate these confounds.

While the majority of the population of microglia that we surveyed in hippocampus exposed to Tat remained in a surveillance phenotype without evidence of transformation into a rod-like, reactive morphology (Figure 3, 4 insets), there was widespread evidence of reciprocal engulfment (Figure 5) of microglia and peripheral myeloid cells, raising the question of whether microglia may attack invading inflammatory leukocytes.

Introduction of Tat into the CNS was also associated with disruption of normal synaptic architecture, as reported previously [28]. Our data cannot unequivocally differentiate the relative contribution of microglia vs. peripheral myeloid cells to phagocytic engulfment of normal synaptic architecture. However, qualitatively, our data in aggregate suggest that microglia do not simply mimic the initial neurotoxic response of infiltrating leukocytes 24 hr after exposure to Tat. While infiltrating inflammatory leukocytes robustly destroy microglia and neuronal dendritic spines in their vicinity (Figure 4B, 9A, green arrowhead), microglia remain largely ramified, albeit with hypertrophied processes (Figure 4, Panels B, D), and appear to ensheath neurons (Figure 7B, 9A, blue arrowheads).

The sequence of signaling events after Tat injection in our model is complex and can be assumed to include release of the chemokine monocyte chemoattractant protein type 1 (MCP-1/CCL2) well as up-regulation of VCAM-1 and ICAM-1, with the ultimate biologic effect of transmigration of mononuclear cells across the BBB into the CNS [43]–[48]. Inflammatory leukocyte infiltration is likely to be further amplified by the ability of Tat to increase the production of platelet-activating factor, PAF, a chemotactic factor for granulocytes [49]–[53]. We do not yet know whether the microglial response to Tat-induced chemotaxis of peripheral inflammatory leukocytes *in vivo* is an attempt at host defense of normal synaptic architecture, but it is interesting to speculate that microglia may attack invading leukocytes, which enter the CNS in response to local neuroinflammation and chemokine release [48], [54], [55]. Microglia are also well recognized for their contribution to the normal “pruning” and maintenance of dendritic architecture [56]. However, in the setting of Tat-induced neuronal damage and neuroinflammation, it is quite possible that their ability to strip synapses may become amplified to a pathologic level - with the result that microglia may elicit enduring neurotoxic responses [57]. Our morphometric data (Figure S2, Panel D) demonstrate a 41% increase in leukocyte and microglial contact with neuronal structures, and when considered in the context of synaptic elements present as inclusions in microglia at 7 d (Figure 10, Panels E, F) and enduring microgliosis at 28 d (Figure 1, Panel D; Figure S1, Panels B, D), this seems like a reasonable supposition to be investigated in future studies.

In addition to demonstrating the rapid infiltration of inflammatory leukocytes into the brain following intracerebral delivery of HIV-1 Tat, our data suggest that the gene dose of CX3CR1 exerts a profound regulatory effect on infiltration of inflammatory leukocytes. Indeed, mice engrafted with homozygous marrow (CX3CR1-GFP^+/+^) had significantly increased numbers of infiltrating mononuclear cells in response to both saline and Tat injection into the brain (Figure 2E, F), when compared with mice engrafted with heterozygous marrow (CX3CR1-GFP^+/−^). This effect was especially striking in mice that received saline alone. In contrast, head shielding had no effect on the magnitude of Tat-mediated leukocyte infiltration into the CNS (Figure S1, Panel B) [37]–[39].

Data in this study as well as a previous study [10] demonstrate that a single injection of Tat into the CNS induces persistent changes in normal neuronal and microglial cellular architecture. While *in vivo* exposure to Tat alone as an experimental model for HIV-1 neuropathogenesis clearly has its limitations, other murine models of HIV-1 infection of the CNS [22], [23], [58] are not compatible with the adoptive transfer methodologies required to dissect the relative contribution of peripheral leukocytes vs. microglia in HIV-1 induced neuroinflammation. Moreover, the profound and durable CNS changes following a single injection of Tat are striking, and speak to the ability of this simple, reductionist model to recapitulate some of the striking features of HAND – including the puzzling persistence of CNS damage in the absence of detectable virus loads.

Here we show that, following a single injection of Tat into the CNS, phagocytic elimination of synaptic structures by microglia persisted for up to 7 days (Fig. 10) and microgliosis persisted for at least 28 days (Figs. 1D and S1), while infiltration of peripheral inflammatory leukocytes was far more ephemeral. Although ephemeral, this transient leukocyte infiltration may be devastating to normal neuronal function. Data from experimental models of stroke and intracerebral hemorrhage suggest that the magnitude of neutrophil and activated monocyte/macrophage infiltration, as well as release of soluble inflammatory mediators are associated with greater neurologic dysfunction [59]–[61].

In conclusion, our data make a compelling case for a sequence of events that are initiated by infiltration of mononuclear and granulocyte subsets of leukocytes in response to the presence of Tat in the CNS. This is associated with the subsequent engulfment of microglial processes and dendritic spines by infiltrating leukocytes and by a microglial response that includes an apparent “attack” on the invading leukocytes. This suggests that peripheral leukocytes and microglia may make quite different contributions to the pathogenesis of brain injury associated with HIV-1 infection of the CNS. The role of microglia in disease may also change over time, since intercellular communication between invading leukocytes and neurons may trigger the release of ‘find me’ signaling molecules such as ATP or UTP [62], [63]. These signals have the potential to trigger a switch to an inflammatory phenotype for microglia – leading to enduring microgliosis and neuroinflammation, long after the initial viral insult has been cleared. Studies in progress are focused on the critical signaling cascades that may be involved in the biphasic microglial response to HIV-induced neuroinflammation.

## Materials and Methods

### Reagents

Samples of Tat_1–72_ were generous gifts from the laboratories of Avi Nath (John Hopkins University). Antibodies for immunocytochemistry were mouse anti-MAP2 (sigma) 1/2000, mouse anti-PSD95 (ABR) 1/500, mouse anti-GFAP (clone GA5, Cell Signaling) 1/1000, mouse anti-MPO (Abcam a16886-50) 1/200, biotinylated mouse anti-GFP (Rockland) 1/5000, rat anti-mouse CD11b (clone 5C6 Serotec) 1/400, rat anti-mouse 1-A/1-E (MHCII) (BD Pharmingen) 1/2000, rabbit anti-GFP (invitrogen A11122) 1/5000 and Alexa 488 conjugated 1/1000, rabbit anti-Iba1 (Wako) 1/1000, and rabbit anti-GFAP (Dako) 1/1000. All the goat secondary antibodies were Alexa dye conjugated highly cross species absorbed (Invitrogen) 1/500. The following antibodies were purchased from BD Bioscience and used for flow cytometry analysis of bone marrow chimeras, CD11b-PE (1∶1000), CD45.2-APC (1∶250), CD8a-PE (1∶1000), B220-PerCP (1∶1000), CD4-PerCP (1∶1000), NK1.1-PE (1∶1000). All other chemicals and reagents were purchased from Sigma (St. Louis, MO).

### Generation of bone marrow chimeras

Bone marrow cells (10×10^6^) from CX_3_CR1-GFP^+/−^ or ^−/−^ mice were injected into the tail veins of lethally irradiated (1000 rad) CD45.1 congenic hosts (or vice-versa). In all cases, the reconstitution efficiency of CD11b^+^ cells exceeded 95% as assessed by flow cytometry at 4 weeks after irradiation by the method of Despande [64]. Subsequent to the original experiments using irradiation chimeras without a head-shield, additional experiments were performed using a modification of this method, because of potential concerns that irradiation of the head might lead to damage to the blood-brain barrier (BBB) [37]–[39]. Specifically, CD45.1 host mice were engrafted with heterozygous CX_3_CR1/GFP^+/−^ bone marrow after receiving 9gv gamma ray irradiation to the whole body with head shielding from the cesium source, which yielded similar data described in the Results section.

### Stereotactic injection Tat into hippocampus, including Ethics Statement

12–14 week-old bone marrow chimeric (4 weeks after engraftment; received bone marrow graft at 8–10 weeks of age) or wild-type C57Bl/6J mice were used in accordance with the NIH guidelines for the responsible use and care of animals under the auspices of the University Committee on Animal Resources (UCAR) at URMC which fully approved this study (PHS Assurance: A-3292-01, Protocol Number: 2008-004). Briefly, mice were anesthetized by i.p. injection of a mixture of ketamine (120 mg/kg) and xylazine (60 mg/kg), and the head firmly secured in a stereotaxic frame, 15 µg Tat_1–72_ in 5 µl PBS or 5 µl PBS was injected into hippocampus of the right hemisphere using stereotaxic coordinates of −1.9 mm posterior to the Bregma (see http://www.mbl.org/atlas170/atlas170_frame.html), 2.3 mm lateral to the midline, and 1.7 mm ventral to the pial surface. A micro-syringe pump (micro4, WPI, Saratoga, FL) and 10 µl Hamilton syringe (33 gauge needle coated inside with Sigmacot, Sigma, St. Louis, MO) were used to deliver 5 µl solution in 10 minutes, followed by an additional 10 minute period prior to needle withdrawal. Mice were sacrificed at 1 d after Tat_1–72_ or control vehicle stereotactic injections for ICC. We also did the Tat treatment in hippocampus of Thy-1 YFP (Jackson Lab), CX3CR1/GFP and C57Bl/6 mice and sacrificed at 8 hrs, 1 d, 2 d, 7 d, and 28 d after Tat_1–72_ or control vehicle injections. Transcardial perfusion of a periodate-lysine-paraformaldehyde fixative (PLP, 3.5% paraformaldehyde) [65] was used to fix the brain tissue.

### Immunocytochemistry

40 µm sections were cut from fixed brains using a vibratome (Leica VT1000S). Immunocytochemical staining with a combination of up to 4 different antibodies, including CD11b, *IBA1*, GFAP, MHCII, MPO, Tuj1, MAP2, and GFP in conjunction with various fluorescent (Alexa 350, 405, 488, 555, 647 or 660 dye [1∶500], Invitrogen) secondary antibodies were incubated using a free floating tissue sections. All primary antibody concentrations were optimized in our laboratory based on pilot experiments with multiple antibody combinations. Sections were mounted on glass slides and coverslips with Prolong antifade media was used, without or with DAPI (Invitrogen), depending on whether an Alexa 350 or 405 secondary antibodies were used to detect the primary antibody.

### Stereotactic injection Tat into cortex and Immunocytochemical EM

12 to14 week-old CX_3_CR1^+/−^ mice received cortical injections of either 9 µg Tat_1–72_ in 3 µl PBS or 3 µl PBS (vehicle control) using a 35 gauge needle with the same microsyringe pump delivering system as described above but at a rate of 80 nl/min. Reagents were delivered to the stereotactic coordinates: −1.0. mm posterior to bregma (see http://www.mbl.org/atlas170/atlas170_frame.html), 1.5 mm lateral to the midline, and 0.8 mm ventral to the pial surface. At sacrifice, mice used for EM analyses were anesthetized with sodium pentobarbital (80 mg/kg, i.p.) and perfused through the aortic arch with 3.5% acrolein followed by 4% paraformaldehyde [66]; groups of animals (n = 3 each) were sacrificed at both 24 hrs and 7 d after Tat or PBS (control) injections. Transverse sections of the brain (50 µm thick) were cut in ice-cooled PBS (0.9% NaCl in 50 mM phosphate buffer, pH 7.4) with a vibratome, immersed in 0.1% sodium borohydride for 30 min at room temperature (RT), washed in PBS, and processed freely floating following a pre-embedding immunoperoxidase protocol previously described [66]. Briefly, sections were rinsed in PBS, pre-incubated for 2 hrs at RT in a blocking solution of PBS containing 5% normal goat serum and 0.5% gelatin, incubated for 48 hrs at RT in rabbit anti-*Iba1* antibody (1∶1,000 in blocking solution; Wako Pure Chemical Industries), and rinsed in PBS. After incubation for 2 hrs at RT in goat anti-rabbit IgGs conjugated to biotin (Jackson Immunoresearch) and with streptavidin-horseradish peroxidase (Jackson Immunoresearch) for 1 hr at RT in blocking solution, labeling was revealed with diaminobenzidine (0.05 mg/ml) and hydrogen peroxide (0.03%) in buffer solution (DAB Peroxidase Substrate Kit; Vector Laboratories). Sections were afterwards post-fixed flat in 1% osmium tetroxide, dehydrated in ascending concentrations of ethanol, treated with propylene oxide, impregnated in Durcupan (Electron Microscopy Sciences) overnight at RT, mounted between ACLAR embedding films (Electron Microscopy Sciences), and cured at 55°C for 48 hrs. Areas located between 2 and 3 mm from the injection site were excised from the embedding films and re-embedded at the tip of resin blocks. Ultrathin (65–80 nm) sections were cut with an ultramicrotome (Reichert Ultracut E), collected on bare square-mesh grids, stained with lead citrate, and examined with a Hitachi 7650 electron microscope.

In each animal, a total surface of ≅1,000 µm^2^ of neuropil was examined. Microglia, neurons, and degenerating neurons were identified according to criteria previously defined [56], [67]–[71]. Degenerating neurons displayed a dark nucleus and cytoplasm, ruffling of the plasma membrane, and extensive invagination of the nuclear membrane, similarly to degenerating neurons in Alzheimer and Huntington disease mouse models and human postmortem brains [67], [70], [71]. In addition to their immunoreactivity for *IBA1*, microglia were distinguished by their distinctive long stretches of endoplasmic reticulm, frequent vacuoles and cellular inclusions, irregular contours with obtuse angles, and associated extracellular space.

### Image analyses

Multi-channel Z-stacks of fluorescence images were taken with a epifluorescence microscope equipped with Opti-Grid structured light illumination that effectively subtracts the out-of-focus fluorescence to achieve a confocal-like image sequence for 3D reconstruction of the spatial relationships among different cell types labeled by antibody immunostaining in the hippocampus. Volocity 3DM (Improvision, Perkin Elmer, Waltham, MA) software suites were used on a Mac G5 computer for image acquisition and analyses. For morphometric quantification of either GFP^+^ or MPO^+^ cell types or cell-to-cell contacts, statistical analyses of data were performed with SPSS software (IBM; http://www.spss.com), and the particular tests used to ascertain significance at the p≤0.05 level are reported in the Results section. To qualitatively assess polymorphonuclear cells (PMNs) infiltration into the CNS, we mounted 1/8 of sections on gelatin-coated slides, stained with H&E, followed by dehydration and coverslipping with permount. A neuropathologist (M.J.), blinded to treatment conditions, then analyzed 4 sections/mouse, from groups of Tat or vehicle treated animals (n = 3 each) for the presence of PMNs present in hippocampus.

## Supporting Information

Figure S1 **Stereotactic injection of Tat induces differential antigen expression in microglia.** This is a montage of hippocampal fields at the injection site 28 d after exposure to vehicle (Panels A, C) or Tat (Panels B, D). Panels A and B depict Iba-1 immunostaining of microglia, while Panels C and D depict the same fields with CD11b immunostaining of these microglia. For Tat and control conditions, n = 3 independent replicates. Scale bar = 20 µm.
(TIF)

Figure S2 **Head-shielding during cranial irradiation does not significantly alter cell counts of Tat-mediated infiltration of leukocytes.** (Panel A); effects of CX3CR1 gene dose on Tat-mediated infiltration of leukocytes (Panel B); effects of Tat on granulocyte infiltration (Panel C) and effects of Tat on volume changes of leukocyte and microglial contacts with neurons (Panel D): In Panels A–D, mice received Tat or vehicle control into hippocampus as described below and were sacrificed 24 hr later. GFP+ or MPO+ cells were counted from 3 sections from each of 3 mice that received Tat or vehicle. The injection site was located in the lateral part of the hippocampus for all the mice treated with Tat or control vehicle, and we counted the total number of GFP+ or MPO+ cells from the whole field of each of three consecutive sections of medial hippocampus separated by 320 µm that were captured using a 10× objective. Total cell counts ± SD for each type of GFP- or MPO-labeled leukocytes for Tat or vehicle control are from 3 independent replicates. For panels A–C, significance was determined by one-way ANOVA with Tukey's HSD posthoc test. For Panel D, we measured the amount of contact between CD11b^+^ structures, i.e. labeled leukocytes and microglia, and YFP^+^ neuronal structures as the intersecting volume between the two from 3D image sets at least 500 µm away from the stereotactic injection site from 4 Tat treated and 3 vehicle treated mice using Volocity 3DM software. Because of the variability of hippocampal YFP expression between mice, we normalized the intersecting volume between CD11b^+^ and YFP^+^ structures by dividing it with the product of total CD11b^+^ volume and total YFP^+^ volume from each individual image set. Here significance was determined by paired T-tests. *^,#^ = P<0.001.
(TIF)
