# Supplementary figures and images for: HIV-1 Tat-Induced Microgliosis and Synaptic Damage via Interactions between Peripheral and Central Myeloid Cells
Source: PLoS One. 2011 Sep 2;6(9):e23915. doi: 10.1371/journal.pone.0023915 (PMC3166280; doi:10.1371/journal.pone.0023915)

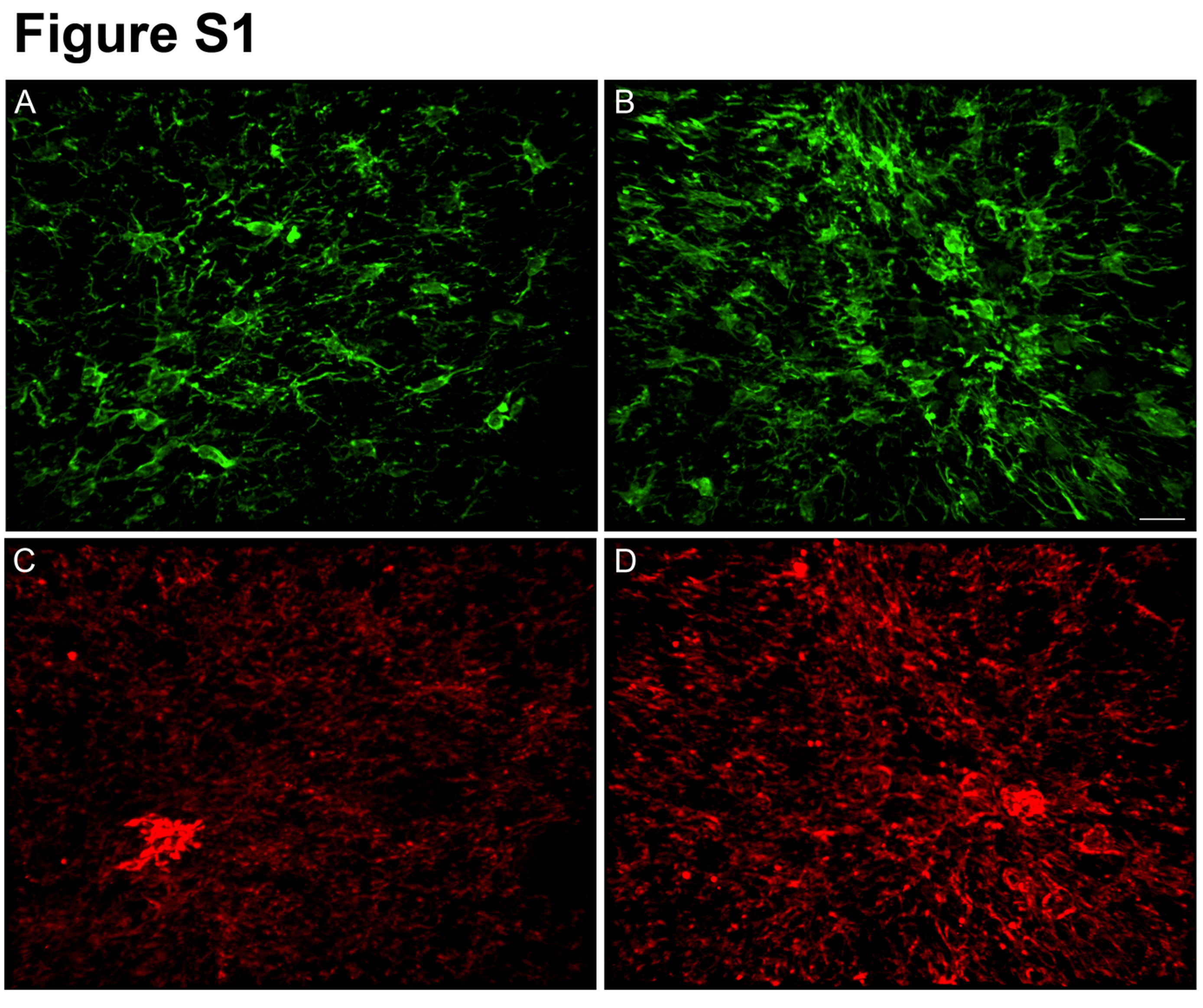

Supplement: Figure S1 — Stereotactic injection of Tat induces differential antigen expression in microglia. This is a montage of hippocampal fields at the injection site 28 d after exposure to vehicle (Panels A, C) or Tat (Panels B, D). Panels A and B depict Iba-1 immunostaining of microglia, while Panels C and D depict the same fields with CD11b immunostaining of these microglia. For Tat and control conditions, n = 3 independent replicates. Scale bar = 20 µm. (TIF) [file pone.0023915.s001.tif]

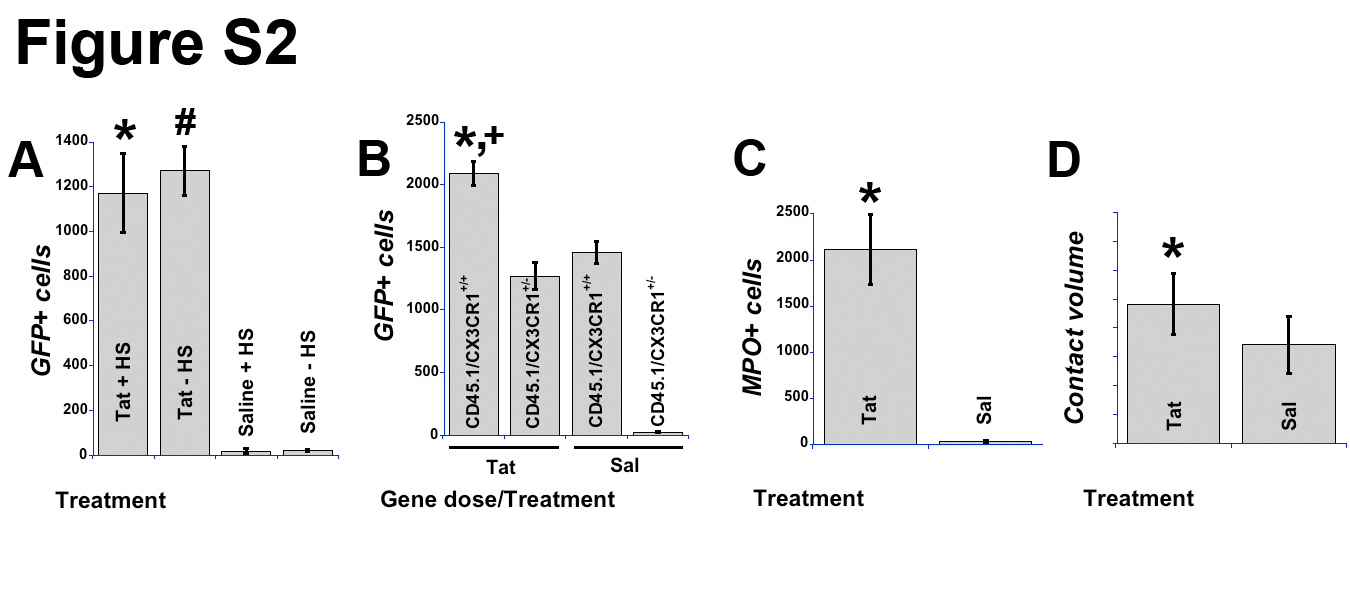

Supplement: Figure S2 — Head-shielding during cranial irradiation does not significantly alter cell counts of Tat-mediated infiltration of leukocytes. (Panel A); effects of CX3CR1 gene dose on Tat-mediated infiltration of leukocytes (Panel B); effects of Tat on granulocyte infiltration (Panel C) and effects of Tat on volume changes of leukocyte and microglial contacts with neurons (Panel D): In Panels A–D, mice received Tat or vehicle control into hippocampus as described below and were sacrificed 24 hr later. GFP+ or MPO+ cells were counted from 3 sections from each of 3 mice that received Tat or vehicle. The injection site was located in the lateral part of the hippocampus for all the mice treated with Tat or control vehicle, and we counted the total number of GFP+ or MPO+ cells from the whole field of each of three consecutive sections of medial hippocampus separated by 320 µm that were captured using a 10× objective. Total cell counts ± SD for each type of GFP- or MPO-labeled leukocytes for Tat or vehicle control are from 3 independent replicates. For panels A–C, significance was determined by one-way ANOVA with Tukey's HSD posthoc test. For Panel D, we measured the amount of contact between CD11b+ structures, i.e. labeled leukocytes and microglia, and YFP+ neuronal structures as the intersecting volume between the two from 3D image sets at least 500 µm away from the stereotactic injection site from 4 Tat treated and 3 vehicle treated mice using Volocity 3DM software. Because of the variability of hippocampal YFP expression between mice, we normalized the intersecting volume between CD11b+ and YFP+ structures by dividing it with the product of total CD11b+ volume and total YFP+ volume from each individual image set. Here significance was determined by paired T-tests. *,# = P<0.001. (TIF) [file pone.0023915.s002.tif]
